# Derivation of Multivariate Syndromic Outcome Metrics for Consistent Testing across Multiple Models of Cervical Spinal Cord Injury in Rats

**DOI:** 10.1371/journal.pone.0059712

**Published:** 2013-03-27

**Authors:** Adam R. Ferguson, Karen-Amanda Irvine, John C. Gensel, Jessica L. Nielson, Amity Lin, Johnathan Ly, Mark R. Segal, Rajiv R. Ratan, Jacqueline C. Bresnahan, Michael S. Beattie

**Affiliations:** 1 Brain and Spinal Injury Center (BASIC), Department of Neurological Surgery, University of California San Francisco, San Francisco, California, United States of America; 2 Department of Comparative Medicine, Stanford University, Palo Alto, California, United States of America; 3 Department of Physiology, Spinal Cord and Brain Injury Research Center (SCoBIRC), University of Kentucky, Lexington, Kentucky, United States of America; 4 Center for Bioinformatics and Molecular Biostatistics, Department of Epidemiology and Biostatistics, University of California San Francisco, San Francisco, California, United States of America; 5 Burke-Cornell Medical Research Institute, Department of Neurology and Neuroscience, Weill Medical College of Cornell University, White Plains, New York, United States of America; University of South Florida, United States of America

## Abstract

Spinal cord injury (SCI) and other neurological disorders involve complex biological and functional changes. Well-characterized preclinical models provide a powerful tool for understanding mechanisms of disease; however managing information produced by experimental models represents a significant challenge for translating findings across research projects and presents a substantial hurdle for translation of novel therapies to humans. In the present work we demonstrate a novel ‘syndromic’ information-processing approach for capitalizing on heterogeneous data from diverse preclinical models of SCI to discover translational outcomes for therapeutic testing. We first built a large, detailed repository of preclinical outcome data from 10 years of basic research on cervical SCI in rats, and then applied multivariate pattern detection techniques to extract features that are conserved across different injury models. We then applied this translational knowledge to derive a data-driven multivariate metric that provides a common ‘ruler’ for comparisons of outcomes across different types of injury (NYU/MASCIS weight drop injuries, Infinite Horizons (IH) injuries, and hemisection injuries). The findings revealed that each individual endpoint provides a different view of the SCI syndrome, and that considering any single outcome measure in isolation provides a misleading, incomplete view of the SCI syndrome. This limitation was overcome by taking a novel multivariate integrative approach for leveraging complex data from preclinical models of neurological disease to identify therapies that target multiple outcomes. We suggest that applying this syndromic approach provides a roadmap for translating therapies for SCI and other complex neurological diseases.

## Introduction

Central nervous system (CNS) trauma evokes complex, interrelated biological and behavioral changes, presenting a major challenge for determining ‘best outcomes’ for assessing translational therapeutic efficacy. Preclinical models provide opportunities to understand the cause-and-effect relationship between functional losses and their underlying biology, however the heterogeneity of CNS trauma remains a barrier for translating findings across different experimental laboratories, injury severities, injury types, and across model species and to humans [1]–[8]. On the other hand, understanding the generality of outcomes in CNS trauma is critical for clinical translation of basic research findings. Integrative methods are required to account for integrated mechanisms of CNS cell death, repair, regeneration and neurological recovery.

To capture the multi-faceted nature of CNS trauma, researchers frequently perform multiple tests on individual subjects. Extensive functional batteries can be found in the literature on traumatic brain injury [9], stroke [10], and spinal cord injury (SCI) [11], [12], among others. Attempts to produce composite ‘neuroscores’ that combine multiple test results exist, however such scales are typically assembled in an arbitrary manner without fully accounting for all inter-relationships among measured endpoints. This is problematic because translational disease features are often reflected in the *association* among outcomes rather than on individual measures [13]–[17]. In this respect, CNS trauma may be viewed as a problem of integrated systems biology, with individual outcome metrics representing individual parts of a holistic syndrome.

Here, we provide a novel approach to measure the ‘syndromic space’ [18], [19] from heterogeneous outcome scales in preclinical models of neurological disorders, using SCI as an illustrating example. We first built an information-rich database containing the total set of detailed behavioral and histological outcomes from 159 rats with various types of experimental cervical SCI and multiple outcome metrics (>15,000 data points). The novel database spanned a range of injury severities, injury modalities (blunt contusion vs. penetrating hemisection), and treatment conditions. Despite data heterogeneity, the large size and detailed nature of the dataset enabled data-driven detection of recovery as a graded, emergent pattern defined within the full multivariate syndromic space. We then went on to test the reliability and validity of our data-driven syndromic approach by evaluating sensitivity to gradations in experimental spinal cord injury, consistency across studies collected over 10 years, and consistency in different injury models (velocity-driven MASCIS/NYU weightdrop vs. force-driven IH, vs. surgical hemisection). The present paper provides proof-of-concept and face-validity of the syndromic approach. Elsewhere, we demonstrate the sensitivity of this approach for the evaluation of different experimental therapies (Ferguson et al.; submitted). The syndromics approach provides a statistical roadmap for translational therapeutic testing of novel therapies for SCI and other neurological disorders.

## Materials and Methods

### Database Development

A large database was constructed by pooling basic cervical SCI data collected at two different institutions (The Ohio State University and University of California, San Francisco), by a group of collaborators over 10 years. Data were highly multivariate; numerous different behavioral and histological measures were collected from the same subjects (24 variables × multiple time-points × 159 subjects, >15900 data points). The goal was to capture the full information about each subject in the database to model the data heterogeneity and diversity of variables collected by the broader SCI field. This included curating all animal care records, detailed biomechanics of injury, detailed histology, and detailed behavioral outcomes into a single database. This approach was analogous to developing an integrated medical record for preclinical studies to enable data-driven translational comparisons at the syndromic level. This database differed from prior data-sharing efforts in SCI in several ways [20], [21]. Prior data-integration work has either focused on a narrower set of measures, for example a single outcome [20], a smaller N [12], [13], or both. The present paper provides a template for expanded multicenter preclinical data-sharing efforts that are currently underway [19]; Nielson et al., submitted).

### Animals

Subjects were 77–87 day old female Long-Evans rats (N = 159) with unilateral cervical spinal cord injuries, using contusion (n = 134), hemisection (n = 9) or sham (n = 16) paradigms. Contusions were produced on one of two different devices: the NYU/MASCIS weight drop impactor (n = 42) or the infinite horizons (IH) force-driven impactor (n = 92). Both devices were fitted with modified impact heads to deliver unilateral injuries. NYU injuries were delivered at 3 injury severities: sham (n = 10), 6.25 mm (n = 10), or 12.5 mm (n = 32). IH injuries were delivered at 3 different severities: sham (n = 6), 75 kdyn (n = 58), or 100 kdyn (n = 34). Hemisections (n = 9) were performed under microscopic control using a number 11 scalpel blade. The goal of including 3 different injury paradigms was to identify consistent multivariate features of cervical SCI that transcend idiosyncrasies of particular contusion devices or injury modalities. A subset of variables (8 of 24) were previously reported in a subset of subjects (n = 31 of 159) as part of an animal model-development paper [22]. Additional information was recovered retrospectively from unpublished ‘file drawer’ records, boosting both the N and variable number [1], [2], [5]. These data were augmented by an additional N = 107 subjects collected prospectively as part of ongoing preclinical trials at UCSF. All experimental protocols adhered to the NIH Guide for the Care and Use of Animals, and were approved by the Institutional Animal Care and Use Committee (IACUC) at The Ohio State University (OSU) and the University of California, San Francisco (UCSF).

### Surgical Procedures for Cervical SCI

All surgical procedures were performed aseptically as described elsewhere [22]. Briefly, animals were anesthetized with Ketamine HCL (80 mg/kg, Abbott Laboratories, North Chicago, IL) and Xylazine (20 mg/kg, TraquidVed, Vedco Inc., St Joseph, MO) i.p. before surgery. A dorsal, midline skin incision was made, the skin dissected and the trapezius muscle was cut just lateral to the midline from C1/2 to T2. Spinous processes from C4 to T1 were exposed and a C5 dorsal laminectomy was performed to expose the entire right side and most of the left side of the underlying spinal cord. Contusion injuries were produced using the MASCIS/NYU injury device [23], [24] with a 10 g impounder dropped from a height of either 6.25 or 12.5 mm, or the Infinite Horizon Impactor (Precision Systems and Instrumentation LLC, Fairfax, VA) with a force of 75 or 100 kdyns. Both impactors were fitted with a modified 2.0-mm-diameter impact head for unilateral injuries. For the hemisection injuries, a #11 scalpel blade was lowered through the entire dorsoventral extent of the spinal cord, without severing the ventral artery. Completeness of the lesion laterally and ventrally was then attempted under microscopic observation using additional cuts with the scalpel blade. The sham control groups underwent the laminectomy procedure without contusion or hemisection. After injury, the wound was closed in layers and the animal recuperated overnight in an incubator. The antibiotic, Cefazolin (50 mg/kg, Henry Schein, Melville, NY) was administered both pre- and post-operatively. All animals were inspected daily for wound healing, weight loss, dehydration, autophagia and any discomfort. Appropriate veterinary care was provided as needed.

### Function: Grooming Test

**Figure 1:**
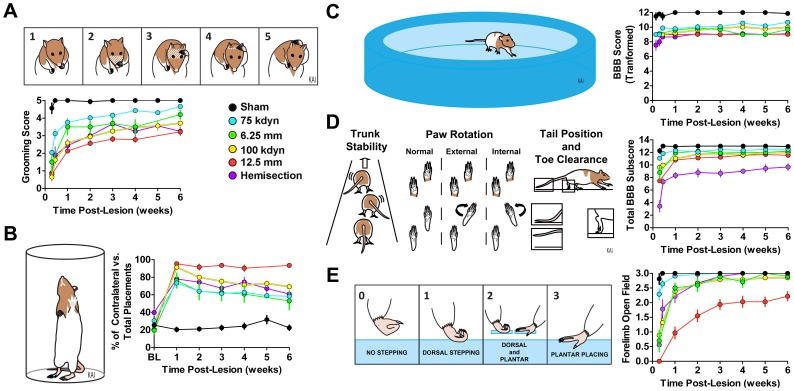
Standardized observation-based behavioral batteries for evaluating recovery after spinal cord injury in the rodents. ***A,*** Grooming scale scoring system and recovery plots color coded by injury conditions. ***B,*** Paw placement task and recovery plots. ***C,*** Basso, Beattie, Bresnahan (BBB) open field hindlimb locomotor scale. ***D,*** Fine motor-focused, BBB subscore. ***E,*** Forelimb open field score. Three different standardized models of SCI were included in the dataset: hemisections, force-driven contusions (kdyns) and weight-drop contusions (mm) centered at cervical vertebra 5 (C5) and delivered to one side of the spinal cord. Data were collected over 10 years at two different SCI centers (The Ohio State University, and University of California, San Francisco) and represent over 159 subjects with complete outcome batteries. Error bars reflect SEM as used by general linear models (e.g., ANOVA). Note that all points have error bars although some are smaller than the points. (see manuscript for references).

Forelimb grooming function was assessed using an adapted grooming test as described in [22]. Cool tap water was applied to the animal’s head and back with soft gauze, and the animal was placed in a clear plastic cylinder (diameter = 20 cm; height = 46 cm). Grooming activity was recorded with a video camera from the onset of grooming through at least two stereotypical grooming sequences (∼2 min). Scoring was performed as illustrated in Fig. 1. Slow-motion video playback was used to score each forelimb independently by the maximal contact made while initiating any part of the grooming sequence. Animals were tested on days 2, 7, 14, 21, 28, and 42 post-operatively.

### Function: Abnormal Paw Placement (Forelimb Asymmetry Test)

Animals were tested in a clear plastic cylinder and spontaneous exploratory behavior was recorded for 5 min. Slow motion video playback was used to determine the number of times the animal placed its left, right, or both forepaws against the side of the cylinder during weight supported movements according to the criteria of [25]. Individual placements were scored as either “left” or “right” when 0.5 sec or more passes without the other limb contacting the side of the cylinder. If both forepaws were used for weight-supported movements within 0.5 sec of each other, a score of “both” was given. Results were reported as a percentage of contralateral limb use versus total paw placements. This measurement technique results in a baseline of about 30%. Animals were tested on days 2, 7, 14, 21, 28, and 42 post-operatively.

### Function: Open Field Locomotion

Hindlimb locomotion was evaluated using the Basso Beattie Bresnahan (BBB) score [11] with a metric transformation that improves score stability [20]. Details of open field performance were measured using the BBB subscore [26]. Forelimb open field was assessed using a simple 4 point scale depicted in Figure 1. Animals were tested on days 2, 7, 14, 21, 28, and 42 post-operatively.

### Function: Automated Gait Analysis

**Figure 2:**
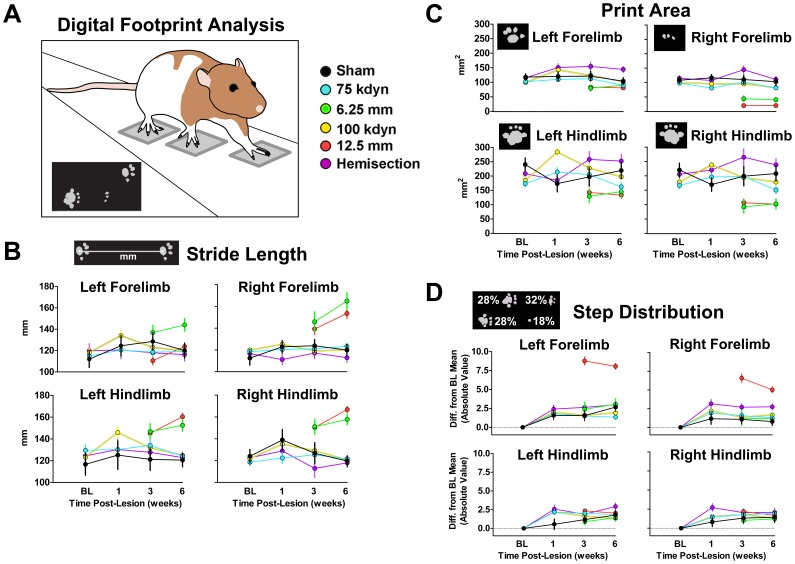
Standardized digital locomotor analysis for evaluating recovery after spinal cord injury in rodents. ***A,*** Digital footprint analysis allows objective quantification of many correlated outcomes including: ***B,*** Stride-length for each limb; ***C,*** Print area for each limb; ***D,*** Distribution of limb use reflected as the absolute deviation from the pre-injury baseline (i.e. deviation from ∼25% recruitment for each limb).

The walkway and CatWalk analysis program were used as described by [27]. Briefly, animals were trained to cross a glass walkway (120 cm long) with black Plexiglass walls (8 cm apart) and ceiling (10 cm from the floor). In a darkened room, light from an encased fluorescent bulb was transmitted through the glass surface of the walkway (0.6 cm thick) and entirely internally reflected within the glass. Paw contact increased reflected light and illuminated the paw print (Fig. 2A) which was collected by a digital video camera underneath the runway. A digital file for each run across the middle 90 cm of the walkway was collected and analyzed using the CatWalk program v.7. Individual digital prints were manually labeled and different quantitative measurements for locomotion were calculated with the software: stride length (the distance between consecutive steps with the same limb); print area during maximal contact; and the distribution of total steps among the four limbs. Before surgery, animals were gently guided to make complete passes from left to right of the walkway. After pre-op training, animals were tested on the walkway at baseline and then 1, 3, and 6 weeks post-operatively. Data were averaged across 5 runs in which the animal maintained a constant speed across the middle 90 cm of the CatWalk runway. Data from week 1 were corrupted and uninterpretable for the NYU/MASCIS injury dataset, so analyses were limited to 3 weeks and 6 weeks post-injury. For the IH and hemisection datasets 1, 3, and 6 weeks were analyzed (see Fig. 2).

### Integration of Functional Metrics

Pre-processing for multivariate integration involved averaging behavioral measures over time to capture temporal variance in a single value. Digital footprint analysis was only collected at week 3 and 6. Therefore these time-points were used for multivariate analysis to ensure no missing values during syndromic pattern detection. Taken in total, behavioral function was represented by 17 outcome measures (Fig. 1–2; Fig. S1).

### Injury/Histological Measures

Six weeks after SCI, animals were sacrificed by anesthetic overdose followed by bilateral thoracotomy and transcardial perfusion with 200 ml phosphate buffered saline (PBS) followed by 500 ml 4% paraformaldehyde. Tissue was post-fixed in 4% paraformaldehyde for <18 h and then processed for either paraffin embedding or cryostat sectioning (cryoprotection in 20% sucrose; blocking in O.C.T. matrix), depending on the study. Tissue was sectioned, and stained with luxol fast blue and cresyl violet. Histological analysis was performed as previously described [22], [28]. Outcomes included sparing measures at lesion epicenter and motor neuron counts throughout the extent of the lesion. All histological measures are expressed as a percentage of sparing, normalized to contralateral control tissue. These same terminal histological outcomes were used to explore the relationship between histology and behavior post-injury. Histology was represented by 6 outcome measures: lesion size, gray matter sparing, white matter sparing, total tissue sparing, total area (spared+cellular), and motor neuron sparing.

### Statistics: Principal Component Analysis (PCA)

Syndromic patterns were detected using PCA by spectral decomposition of the cross-correlation matrix of all outcomes [29] using the FACTOR subcommand with multiple imputation for sparse missingness in SPSSv.19. This method essentially detects consistent patterns within complex systems of inter-related variables. In the case of neurological disorders, robust inter-relationship among variables can be interpreted as statistically sound measures of the complete syndrome of SCI as measured by the composite set of outcomes metrics (forelimb function, hind limb function, and histology). Mathematically, PCA extracts composite, synthetic variables known as principal components (PCs) that reflect uncorrelated (orthogonal) partitions of shared variance. It is common practice to rotate the factors for the purposes of interpretation. The most common rotation method, varimax, retains the orthogonal nature of the PCA. However it has been noted that under certain circumstances multivariate patterns are correlated and orthogonal PCs are artificial representation of the data [30]. In the case of SCI, PC orthogonality could be problematic because syndromic measurement may reflect distinct functional states that are nonetheless interrelated. For example, weight-supported stepping may be detected as one PC and coordination as another. Although these functional states may be measured by different sets of variables, their recovery is nonetheless correlated. The method of oblique rotation allows testing for PC correlations. Pilot analyses did not reveal fundamental differences between initial extractions, varimax, or oblique rotations, indicating that syndromic measures PC1-3 are truly orthogonal (data not shown). PCs were retained using 4 criteria: 1) the Kaiser rule, retaining PCs with eigenvalues >1.0 [31], 2) Scree plot [32], and 3) the over-determination of the factors [33], retaining factors with at least 3 loadings above |.4|. PCs meeting all three criteria were examined and named using loadings above |.4|, thereby accounting for at least 20% of the variance (see Figs. S1, S2, S3, S4).

### Validity Analysis

To assess the validity of syndromic patterns we performed 3 tiers of analysis. First, we used the standard factor analytic approach of examining loading patterns for face validity: do the PC patterns reflect coherent clusters of variables and can they be given names? Second, we analyzed content validity by evaluating whether PCs had high loadings from key histological and behavioral variables used as ‘gold standards’ by the SCI field. Third, we used analysis of variance (ANOVA) to test the sensitivity of PC scores to injury gradations. ANOVAs were performed using the SPSS GLM subcommand on PC scores extracted by PCA. Significant ANOVAs were followed by Tukey’s posthocs. Significance was assessed at p<.05.

### Construct Validity and Reliability: Permutations, Feature Sub-selection and Cross-Validation

Construct validity is the concept that syndromic patterns reflect true underlying states rather than mere artifacts of the variables collected. In the context of SCI such constructs could include states such as ‘neuroprotection’ or ‘recovery of coordination’ which are hypothesized to manifest across a broad range of specific outcome variables such as ‘BBB score’, etc. Construct validity can be assessed by comparing the consistency of PC loading structure across different subpopulations of variables. Syndromic measures with high construct validity will demonstrate consistent loading patterns and high communalities, independent of the subset of outcome variables collected. In addition, robust syndromic measures should remain consistent across different subpopulations of subjects, a concept known as cross-validation. Cross-validation can be taken as a measure of predictive validity: the ability of syndromic patterns to generalize across different subject populations. Comparisons of the loading patterns across different PCA extractions were performed using the root mean squared difference in PC loadings (RMS), the coefficient of congruence (CC), the Pearson product moment correlation coefficient (r); and the salient variable similarity index (s); [34]. Each of these statistics represents a distinct computational strategy; however, Monte Carlo studies suggest that they yield similar results with consensus of the 4 statistics providing a sound criterion for assessing replication of syndromic patterns [35]. Although there are no established cutoffs for significance of the RMS and CC, researchers have suggested that RMS values approaching 0.0 and CC approaching 1.0 [36] indicate an adequate fit of both the sign and the magnitude of the pattern loadings. For the present study, PC pattern agreement was set at p<.05 for both Pearson r and the salient variable similarity index, s. For s, we adopted the conservative cutoff of |.4| for assessing salient loadings [33]. We augmented these analyses with application of a recently published sparse PCA algorithm that uses an L1 penalty (via the lasso/elastic net approach) to simultaneously perform shrinkage of PC loadings and feature (outcome variable) subselection [37]). Sparse PCA was performed using package PMA (version 1.0.8) under R (version 2.15.2). Consensus of these permutation analyses was interpreted as evidence of syndromic measurement consistency.

## Results

**Figure 3:**
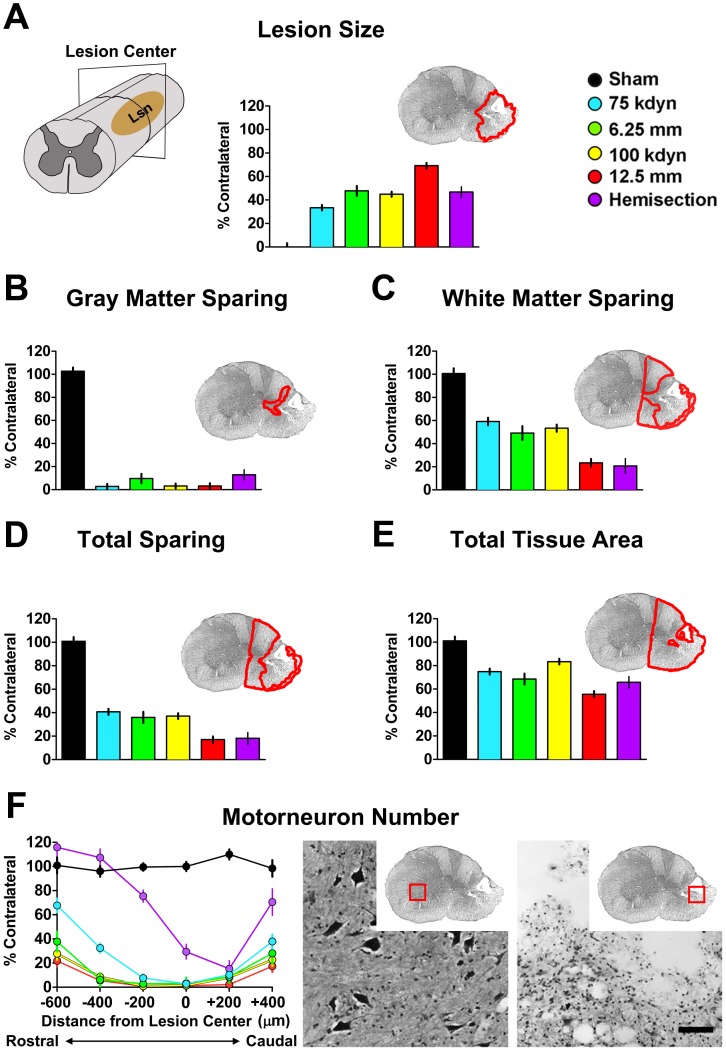
Histological outcomes. ***A,*** Tissue sparing measures in SCI research are typically taken at the lesion center as determined by the largest extent of the lesion ellipsoid. Although specific methods for quantification may vary across studies, typical measures include lesion size, ***B,*** gray matter (GM) sparing, ***C,*** white matter (WM) sparing, ***D,*** total sparing (GM+WM), ***E,*** total tissue area (GM+WM+debris), ***F,*** motorneuron number. Scale bar, 100 µm. Since the compiled dataset was limited to unilateral injuries (hemisections or hemicontusions), all measures are represented as a percentage of the contralateral, spared hemicord. The quantified area is illustrated in red on a representative example. The representative example was taken from the subject closest to the group mean for lesion size across the study’s 159 subjects.

**Figure 4:**
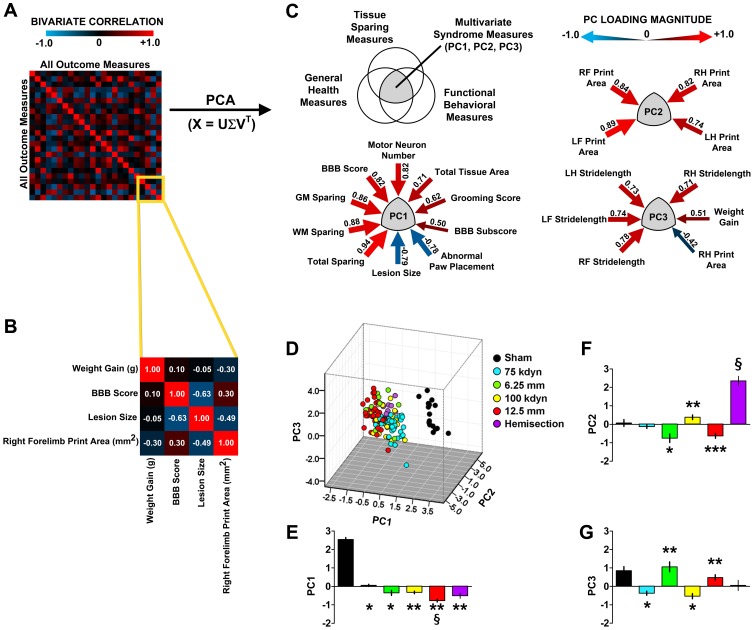
Multivariate analysis of the SCI syndrome using data from two research sites. ***A,*** Heat map of the bivariate correlation matrix, indicating all cross-correlations between behavioral and histological outcomes sorted in a randomized fashion. Blue indicates negative relationships and red indicates positive relationships. Heat reflects magnitude of Pearson correlation (r). ***B,*** Zoomed view of a small portion of the correlation matrix showing the interrelationships between a subset of outcomes. ***C,*** Principal components analysis (PCA) by eigenvalue decomposition was used to reduce the correlation matrix to synthetic multivariate variables known as principal components (PCs). PCs reflect clustered variance shared by numerous outcome measures. PC identities are indicated by significant PC loadings (arrows, loadings |>.40|). Each loading is equivalent to a Pearson correlation between individual outcomes and the PC. Loading magnitude is indicated by arrow width and heat (blue reflects negative and red reflects positive relationships). Exact loading values are shown next to each arrow. See Fig. S1 for non-significant loadings. ***D,*** Plot of individual subjects (N = 159) in the 3D multivariate syndrome space described by PC1-3. ***E–G***, 2D plots of PC1-3 on their own axes. Significant differences: E,*P<.05 from sham, ** *P*<.05 from 75 kdyn and sham, §*P*<.05 from all groups except 6.25 mm. ***F,*** *P<.05 from sham, **P<.05 from all groups but sham, ***P<.05 from sham, 75 kdyn, 100 kdyn and hemisection. §*P*<.05 from all other groups. ***G,*** *P<.05 from sham, ** *P*<.05 from 75 and 100 kdyn.

**Figure 5:**
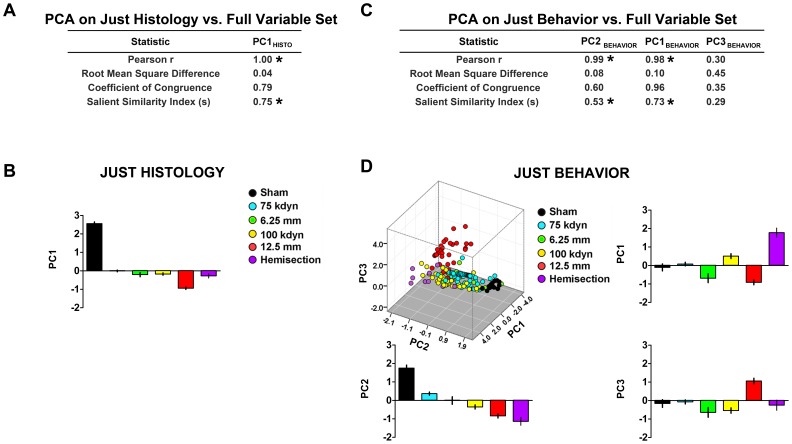
Consistency of PCA across subsets of variables. ***A,*** Independent PCA extraction using only histological variables demonstrated significant replication of PC1 extracted using the full variable set. ***B,*** Injury condition affected PC1_HISTO_ in an equivalent manner to the full-variable extraction (compare to Figure 4E). ***C,*** PCA extraction using only behavioral variables significantly replicated the full variable PC for PC1 and PC2, however the sequence of extraction reversed, indicating a reversal in variance explained by PC1_BEHAVIOR_ and PC2_BEHAVIOR_. ***D,*** Scores of individual subjects on PC1-3 extracted from just-behavioral variables. The pattern for PC2_ BEHAVIOR_ recapitulated PC1 from the full variable extraction (compare to Fig. 4E) and PC1_BEHAVIOR_ recapitulated PC2 from the full-variable extraction (compare to Fig. 4F). *P<.05 for replication statistics, s>0.63.

**Figure 6:**
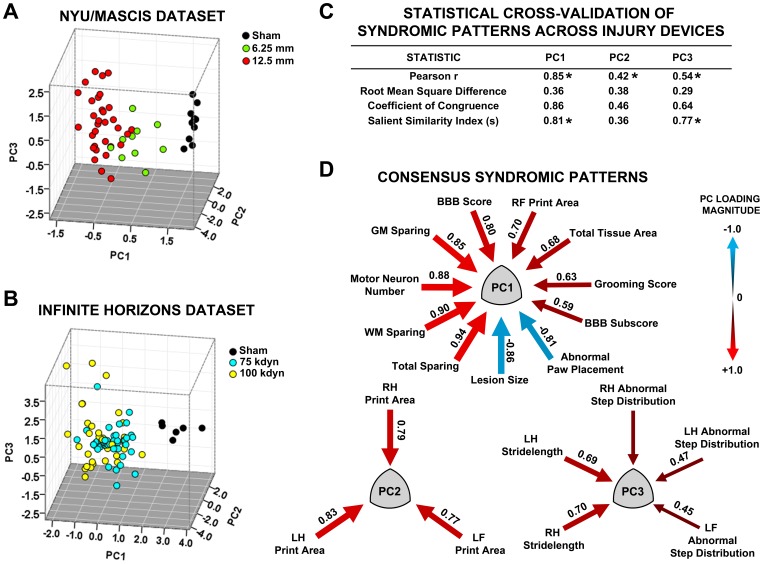
Consistency of multivariate syndromic patterns across two different biomechanically controlled cervical spinal contusion models. ***A,*** SCI syndromic space extracted from an NYU/MASCIS injury device dataset (N = 52 rats; 24 outcome variables). ***B,*** SCI syndromic space extracted from an Infinite Horizons injury device dataset (N = 100 rats, 24 outcome variables). Note, normed PC score axes are scaled according to variance within each extraction, resulting in axes with units that are not directly comparable across extractions. However relative relationships among groups (sham vs. injuries) are conserved. ***C,*** Consensus PC loading patterns that are conserved across injury patterns. Loading weights (arrows) reflect average values across the two datasets. ***D,*** Statistical evaluation of PC cross-validation in the PC loading matrices from NYU/MASCIS and IH injury datasets. *p<.05 for n = 24 variables; s>0.63.

Comparisons across injury models are described in both univariate (Fig. 1, 2, 3) and multivariate (Fig. 4, 5, 6) analyses. Our approach was to incorporate all information we could gather for each subject. Our goal for data annotation/curation was to achieve complete granularity of measured outcomes and minimize assumptions/human interpretation whenever possible. Toward this end, data were collected in a blinded fashion during initial acquisition and data-driven analyses were performed blind-to-condition. The goal for this approach is to provide unbiased data to fuel data-driven multivariate pattern detection of the entire SCI syndrome. In this context, the extent to which metrics representing syndromic patterns respond to graded injury provides a powerful test of validity.

### Observational Behavioral Scales Show Graded Recovery across Different Cervical Spinal Cord Injury Types and Severities

To evaluate the SCI syndrome, subjects were given a C5 unilateral SCI followed by outcome-monitoring by blinded raters using a battery of observational tests of forelimb use (Fig. 1A–B) and locomotor function (Fig. 1C–E). Subjects (N = 159) were evaluated for 6 weeks, the typical monitoring window within the SCI literature [38]. By pooling data, we achieved higher N’s than typically seen in experimental SCI research, resulting in smaller standard errors (error bars in Fig. 1, 2, 3), higher-power for detecting injury severity (1-β range: 0.935-1) and recovery over time (1-β range: 0.99-1). Different behavioral scales resulted in slightly different rank-ordering for injury severities, with the worst performance with either 12.5 mm weight drop contusion or hemisection, depending on the scale (e.g., Fig. 1D versus E). This suggests that different measures are differentially sensitive to biological mechanisms of injury and repair. The findings indicate that each measure provides a different view of the SCI syndrome, and that considering any single outcome measure in isolation provides an incomplete view of the SCI syndrome.

### Automated Digital Locomotor Analysis of Recovery across Different Injury Types and Severities

To capture fine locomotor changes we used an automated digital footprint analysis system that provides quantitative information on limb recruitment pattern during locomotion across a transparent runway (Fig. 2A) [22]. Measures for each limb included linear distance between consecutive steps (stride-length) (Fig. 2B), print area (Fig. 2C), and proportional limb recruitment (step distribution; Fig. 2D). Footprint analysis is thought to be more sensitive to fine-motor coordination than observational batteries [39]. As with observation-based batteries, the different forms of digital footprint analysis produced different rank ordering of the injury groups. This suggests that the various gait measures may reflect distinct biological mechanisms. For example, some measures may detect neuroprotective changes associated with cellular sparing whereas others may reflect endogenous plasticity associated with neurite outgrowth, for example. It should be noted however, that using any single measure in isolation, provides an inaccurate view of the SCI syndromic state. Moreover, resolving mechanistic effects requires integration of behavioral data with histological data.

### Histological Changes across Different Injury Types and Severities

Histopathology was performed at 6 weeks post-injury as previously described [22], [28], [40]. The lesion center was defined as the coronal section with the largest cross-sectional lesion area in camera lucida drawings of Luxol fast blue/Nissl-stained sections (Fig. 3A). A number of anatomical measures were taken at the lesion center, including gray matter sparing (Fig. 3B), white matter sparing (Fig. 3C), total sparing (Fig. 3D), total tissue area (Fig. 3E) and motorneuron sparing (Fig. 3F). All measures were normalized to the contralateral, uninjured tissue [22]. The chosen histological measures have been used as ‘gold-standards’ to evaluate therapeutics that target sparing of neurons and white matter [40], [41], and are often augmented by more detailed immunomarkers for specific cellular elements [28], [42]–[44]. Claims of therapeutic benefit within the preclinical SCI literature are typically based, at least in part, on the use of these histological markers, albeit sometimes with different quantification methodologies [19], [23], [28], [44]–[48].

### Integrative Multivariate Analysis of Injury Type and Severity

Syndromic pattern-detection of SCI was performed using principal components analysis (PCA). PCA is a classic method for linearly transforming a set of multivariate observations into a set of uncorrelated variables (the principal components) such that the first principal component has maximal variance, the second has maximal variance subject to being orthogonal to the first, and so on [29]. Well-established principal component (PC) extraction and retention rules (eigenvalue>1 [31]; Fig. S1A), scree plot [32]; Fig. S1B), and factor over-determination [33]; Figure. S1C) indicated that the first 3 PCs represented robust clustering of variance of general health outcomes, histopathology, and behavioral functions (Fig. 4A–C). Analysis of PC loadings indicated that PC1 reflects the association of histological sparing variables and behavioral recovery variables, accounting for 34.5% of the variance in the dataset. PC2 (21% of the variance), reflects fine motor control as detected by print area with substantial contributions (loading>0.3) for stride-length and BBB subscore (see Fig. 4C). PC3 (7.68% of the variance) reflects the relationship between weight gain and increased stride-length on the CatWalk.

### Validity of Syndromic Patterns

To validate the syndromic patterns produced by PCA, we tested their sensitivity to the effects of graded spinal cord injuries. We first projected each subject’s position in the SCI syndrome space using standardized PC scores (4D; Movie S1) and tested the effect of injury severity and injury type by ANOVA. SCI severity had a large effect on PC1 (*P*<0.05; Fig. 4E), with 12.5 mm weight drop as the most severe injury, followed by hemisection and the 2 levels of force-driven (IH device) contusions. PC2 was highly sensitive to the effects of hemisection (*P*<0.05; Fig. 4F), distinguishing this injury modality from contusive injuries. Both PC2 and PC3 appeared to detect differences in the weight drop (NYU/MASCIS device) from force driven (IH device) contusion injuries (*P*<0.05; Fig. 4G), suggesting biological differences may exist across different contusive injury devices.

### Syndromic Effects of Cervical Contusion Injury Device

The specific biomechanical factors responsible for differences between impactors are unclear, as the two devices control different aspects of injury. The NYU/MASCIS device delivers a standardized velocity (dictated by gravity); force, tissue compression, and compression rate are all free to vary as a function of velocity [24]. The IH device, on the other hand, uses a servo-feedback motor to deliver a standardized force, however the velocity, tissue compression and compression rate vary during the impact [49]. In addition, the two devices differ in their control of ‘dwell time’ in the fully compressed position. The IH device delivers a standardized dwell whereas the NYU/MASCIS delivers a variable dwell.

The fact that the extent of tissue displacement is not directly manipulated, and varies as a function of other parameters for both NYU/MASCIS and the IH device, suggests that displacement provides a common metric for comparing the biomechanics of the devices. Using covariance analysis we were able to statistically test the hypothesis that variance in tissue displacement dictates the observed differences on the syndromic measures represented by PC1-3. We first used a simple transformation to standardize the displacement units across the injury devices, converting mm (NYU output) to microns (IH output). We then used this new variable ‘standardized tissue displacement’ in analysis of covariance (ANCOVA) to correct for variance in standardized displacement and re-evaluated the effects of injury device. ANCOVA revealed that standardized tissue displacement was a significant covariate of PC1 (p<0.001) but not PC2 and PC3 (p>0.05). However, correcting for tissue displacement had little appreciable effect on the syndromic injury (Figure S2). The effect of intended injury level on PC1 outcome remained statistically significant after correcting for the extent of tissue displacement, all p<0.001. This suggests that the degree of tissue displacement is not the only variable dictating the degree of injury. We therefore can consider ‘intention to treat’ gradations in injury severity the best predictor of individuals’ positions in the full multivariate syndromic space. Moreover, we can conclude that PCA detects syndromic features that have high face-validity with respect to injury severity as it is highly sensitive to the effects of injury gradation.

### Reliability and Construct Validity of Syndromic Patterns: Cross-validation across Outcome Subsets and Sparse PCA

To verify that PC scores represented stable syndromic metrics, we performed two types of statistical perturbation analyses: 1) outcome sub-selection to test the impact of different measurement variables on PC stability and 2) case sub-selection to test for replication and cross-validation of PCs across different subpopulations of individuals. To perform outcome subselection, separate PCAs were performed on histology alone, or functional variables alone and the resulting PC loadings were compared to the full-variable extraction using factor pattern matching statistics on PC loading matrices (Fig. 5). PCA on histological variables alone produced a single PC that significantly replicated PC1 from the full-variable extraction (Fig. 5A–B). PCA on behavioral variables alone, produced a different sequence of PC extraction from the full extraction, with PC2_BEHAVIOR_ replicating the identity of PC1 from the full extraction and PC1_BEHAVIOR_, replicating the PC2 from the full extraction (Fig. 5C–D). What this means is that the the rank ordering of the variance explained by the first two PCs reversed when histology was omitted, resulting in PC1 becoming PC2 and vice versa. Nevertheless, the fundamental syndromic patterns captured in the first 2 PCs remained consistent. PC3_BEHAVIOR_ did not significantly replicate results from the full-variable PCA. Together the results indicate that PC1-2 reflect consistent syndromic measures independent of the variables used to detect the multivariate syndrome with slight changes in the relative proportion of the variance captured by each PC. This strongly suggests an internally consistent SCI syndrome that demonstrates construct validity on PC1-2, independent of the outcome variables collected. The implications of this finding include: 1) there may be efficient means of defining the SCI syndrome that can serve as metrics for evaluating treatments, 2) that these syndromic outcome metrics are more stable than individual outcome variables for detecting the effect of injury severity and recovery of function after SCI, and 3) that syndromic outcomes enable robust comparisons across diverse injury models.

### External Validity of Syndromic Patterns: Cross-validation across Contusion Injury Devices

To use syndromic analysis for translational comparison requires measurement consistency across heterogeneous datasets. To test this in the current database we performed separate subpopulation analysis on NYU/MASCIS vs. Infinite Horizons impact device injuries and then compared the syndromic patterns (Figure 6). The general directionality of the syndromic space was consistent across the subpopulations of NYU/MASCIS and IH injuries: shams had higher scores on PC1 in both datasets, and more severe injuries had lower scores (Fig. 6A–B). The axes have different scales for the different PC extractions, however it should be noted that this should not be interpreted as differences across the devices as PC extractions are standardized to the internal variance structure (mean centered at 0, with SD = 1) for each dataset. To test whether the fundamental PC patterns were conserved across the different injury models we analyzed the PC loading patterns using pattern matching statistics (Fig. 6C). PC1 and PC3 were complete matches across the two different injury models. PC2 was a partial match; however the PC consistency did not reach significance on all pattern matching statistics. Examination of the PC2 loadings indicates that PC2 varied in the loading of the right forelimb print area and stride-length measures across the two different injury devices. The full loading matrices for the extractions from the two different devices are shown in supplemental material (Fig. S3–S4). To visualize the consensus of the syndromic patterns across devices, we generated a consensus matrix containing only loadings that were above |0.4| in both datasets. These significant loadings were then averaged across the two datasets and converted into path diagrams (Fig. 6D), demonstrating clear consensus across the two devices.

Another question that arises is what are the N requirements for detecting syndromic pattern? PCA is essentially a descriptive statistical approach that discovers whatever patterns exist in the data, given the current data. In this sense there is no real limitation in the N. The real concern is whether the detected patterns will generalize to new data, and data collected in typical laboratory studies with smaller Ns. To assess this, we performed 2 additional analysis: 1) a random subsampling procedure to homogenize group sizes across injuries prior to PCA extraction, and 2) a sparse PCA method with an L1 penalty that simultaneously performs variable subselection and shrinkage of PC loadings to assess robust cross-validation [37]. The results of these analyses indicate that the SCI syndromic patterns are, by and large, consistent for the 3 PCs (Figure S5). In particular, PC1-3 remained robust across equalized group sample sizes (n = 9) for 10 different randomized samples (Figure S5A). Sparse PCA (SPCA) verified robustness of PC1-3, however the L1 penalty altered the proportion of the variance explained by some of the PCs, demoting some and promoting others (Figure S5B–C). In addition, SPCA generated drop out of the loadings for some of the individual variables, corroborating the concept that the integrated syndromic patterns were more robust predictors than single variables.

Together the statistical perturbation analyses suggest that these syndromic patterns represent robust underlying disease states that translate across different types of research data. An integrated combination of histological and behavioral variables could therefore provide a new valuable metric for therapeutic testing that is more sensitive, more reliable, and more comprehensive than scores on any one single variable. The application of the syndromic approach to preclinical therapeutic testing and cross-species translation is the topic of ongoing work.

## Discussion

The present paper describes a systems biology approach to define the SCI syndromic space from basic preclinical laboratory research. Data were consolidated from several projects into a single database that combined detailed behavioral and histological data yielding >15,000 data-points from 159 subjects with cervical SCI. Data-driven statistical pattern detection revealed 3 orthogonal syndromic measures (PC1–PC3) that were highly sensitive to gradations in injury severity and injury modality (contusion vs. hemisection), providing a consolidated syndromic space for integrative therapeutic testing.

Univariate analyses of the preclinical database revealed an essentially random pattern of the rank ordering of individual outcome variables according to injury severity, indicating that the most systematic effects were observed at the multivariate level. These data suggest that univariate analysis (e.g., bivariate correlation/regression, ANOVA, t-tests), used by many fields in preclinical research, have the potential to produce inconsistent ‘votes’ for the significance of experimental effects depending on which outcome variables are analyzed. The inconsistencies at the univariate level may contribute to both type I errors (reporting therapeutic benefit when there is none) and type II errors (reporting no therapeutic effect when one indeed exists). In the context of therapeutic testing in neurobiological disease models, these sources of statistical wobble are likely contributors to failures in replication and clinical translation.

Particularly troubling is the fact that univariate significance testing of multiple inter-related outcomes has the potential to promote publication of results that are spurious, or at least, idiosyncratic to a particular set of outcomes. What this means for the taxonomy of neurological disease is that individual outcome measures cannot provide a complete picture of the larger syndrome, limiting replication of findings. As a general example, in the present paper we found that simple measures of functional performance–grooming and paw preference–were highly predictive of total tissue sparing. However fine-grained functional performance measures of gait did not predict histological sparing in a reliable fashion, suggesting that these measures tap into subtle biology that was not represented by tissue sparing markers. At the univariate level this distinction in variable clustering was not obvious, with occasional spurious correlation emerging between gait measures and histology, at a below-chance rate of (17/64). If a researcher had made the decision to use gait parameters as a primary readout for a neuroprotective therapy, it is likely that the data would yield a null finding, whereas a different measure such as paw placement may have yielded a positive effect. Such a scenario could lead to the failure to detect a positive therapeutic effect that does indeed exist, and lead to inconsistent replication of findings. Since replication is a critical step in translational testing, such findings represent a stumbling block for translational therapeutic development [50]. NIH-NINDS has funded 3 major replication centers for SCI in the United States to attempt independent replication of high-profile preclinical therapies. To date, the majority of these replication attempts have been unsuccessful [3], [4], [6]–[8] even though the initial studies were published in high-profile journals [51]–[55] (for exception see [56], [57]). As SCI and other preclinical fields struggle to translate basic research findings to disease syndromes in humans, it may become essential to pool bio-behavioral outcome data across studies to make novel preclinical syndromic discoveries. In other fields with complex information (e.g., experimental physics, economics, sociology, psychology, epidemiology, and genomics), databases are standard tools. Mining of raw data from publicly-funded preclinical research has great potential to allow useful knowledge to be extracted from the large repository of published information and information that currently resides, unpublished and unseen, in laboratory file drawers worldwide.

The present paper demonstrates the feasibility of large scale data-mining from paper and digital records using pooled bio-behavioral data from basic SCI studies spanning 10 years. The findings reveal that experimental cervical spinal cord injury is a multivariate syndrome that is best characterized as a *pattern* across numerous, inter-related mechanistic outcomes. This conclusion, in retrospect, is not surprising, and is consistent with prior work that has proposed combinatorial therapeutics to target multiple mechanisms of the injury cascade [58]. However there have been only a few examples of combinatorial measurement of outcomes at the multivariate level [13], [14], [17] and to our knowledge there have been no studies that have performed large-scale integrative analysis with large numbers of preclinical subjects. This opens the possibility that the field may have missed critical findings because of a lack of multivariate monitoring of outcome. It is possible that hidden cures that have already been discovered have been missed because the field simply lacked the analytical tools to recognize them.

It is important to understand that multivariate approaches can produce unstable results unless applied to large sample sizes or with special statistical safeguards (e.g., lasso penalty [59]). Most basic SCI laboratories do not have dedicated resources for producing datasets as large as the one reported here. For this reason, we have begun a collaborative data-sharing project with 8 major SCI research centers in the United States. Our goal is to help define common data elements (CDE) for the basic SCI field and build a data repository for large-scale contributions from other research groups. In future work, researchers with more modest N’s will be able to leverage the collective knowledge of the field by comparing their data to large databases of syndromic outcomes such as those presented in the present paper. CDE efforts are underway in the clinical realm for SCI and traumatic brain injury [60]–[62]. In addition the clinical SCI field is developing novel grading schemes to integrate outcome and imaging data. The present work represents a parallel effort for the preclinical realm to provide a framework for translational informatics in the coming years. Our platform is designed for expansion to include multiple additional measures of biological and functional outcomes, for example, biomechanical features of injury (e.g., angle; impact head geometry) [63], biochemical tests of inflammatory cytokine production and cellular changes (e.g. microglial activation) related to the TNF cascade [28], [64]. Larger scale data mining is likely to reveal other common syndromic components of SCI that transcend injury severity, species, and mode of injury. The present work provides proof-of-concept by identifying principal components that are shared across a range of injury severities (i.e. from mild contusion to transection of the cervical hemicord). Testing the generality of syndromic measures across novel datasets remains an area for future research.

By providing a community resource for neurotrauma researchers we hope to help catalyze understanding of the proximal molecular, cellular, and system drivers of distinct, complex behavioral outcomes in spinal cord injury. Such information will continue to provide new opportunities to focus and identify new hypotheses for therapeutic intervention that hopefully will ultimately increase success rates and replication in animal testing and ultimately translation to humans.

## Supporting Information

Figure S1 **Decision rules for PC retention and interpretation in complete dataset including cervical hemisection and hemicontusions.** ***A,*** PCA performed on the full dataset (N = 159, 24 outcome variables) revealed 5 PCs that met the liberal ‘Kaiser rule’ criterion of eigenvalue >1. ***B,*** Conservative, scree plot criterion suggests retaining 3 PCs for interpretation. ***C,*** Factor over-determination criterion suggests that the first 3 PCs capture a substantial portion of the variance (loading >0.4 in more than 3 outcome variables), suggesting that the first 3 PCs are stable and represent interpretable syndromic features.
(TIF)

Figure S2 **Effects of injury severity on PC1-3 after correcting for tissue displacement.** Analysis of covariance (ANCOVA) indicated that tissue displacement was a significant covariate, for PC1 p<.05. However, correcting for tissue displacement did not alter the statistical significance of injury effects (compare to Fig. 4E–G). This suggests that differences across the injury devices were multivariate in nature and correcting for the biomechanical feature of displacement did not statistically account for the effects of injury.
(TIF)

Figure S3 **Decision rules for PC retention and interpretation after NYU/MASCIS injury.** ***A,*** PCA performed on sham controls and graded cervical hemicontusion injury performed with the NYU/MASCIS weight drop (N = 52, 24 outcome variables) revealed 5 PCs that met the liberal ‘Kaiser rule’ criterion of eigenvalue >1. ***B,*** Conservative scree plot criterion suggests retaining 3 PCs for interpretation. ***C,*** Factor over-determination criterion suggests that the first 3 PCs capture a substantial portion of the variance (loading >0.4 on >3 outcome variables). Together the decision rules indicate that the first 3 PCs reflect stable and interpretable syndromic features.
(TIF)

Figure S4 **Decision rules for PC retention and interpretation after IH injury.** ***A,*** PCA performed on cervical hemicontusion injuries with the IH device and sham controls (N = 100, 24 outcome variables) revealed 5 PCs that met the liberal ‘Kaiser rule’ criterion of eigenvalue >1. ***B,*** Conservative, scree plot criterion suggests retaining 3 PCs for interpretation. ***C,*** Factor over-determination criterion suggests that the first 3 PCs capture a substantial portion of the variance (loading >0.4 on >3 outcome variables), indicating that the first 3 PCs reflect stable and interpretable syndromic features.
(TIF)

Figure S5 **Cross-validation exercises using equalized n across groups and application of sparse PCA.** ***A,*** Results from an iterative subsampling procedure used to homogenize group sizes (n = 9/injury condition) prior to PCA through 10 randomized subsampling iterations. PC pattern matching statistics comparing subsampled PC loading patterns to the loading pattern from the original dataset were averaged across iterations, revealing significant PC consensus in the subsampled populations. ***B–C,*** Application of a sparse PC algorithm with an L1 penalty to further evaluate PC consistency. ***B,*** Profile of cross-validated sums-of-squares errors as a function of extent of penalization suggested using a penalty value of 3. ***C,*** Sparse PC (SPC) loading matrix after penalty-induced shrinkage toward 0. Blanks indicate loadings of 0. Note that SPCA demoted PC1 moving it to PC3. Further examination of SPCA vs. other modern algorithms will be the subject of future *in silico* work using federated databases of SCI data that are currently under development.
(TIF)

Movie S1 **Multiple views of the multivariate syndromic space characterized by PC1-3.** Each subject is represented as a unique point within the syndrome space. Note that each PC axis is orthogonal to the other axes, indicating that differences in the syndrome features characterized by PC1 are independent from differences along the PC2 and PC3.
(AVI)
