# Supplementary figures and images for: Derivation of Multivariate Syndromic Outcome Metrics for Consistent Testing across Multiple Models of Cervical Spinal Cord Injury in Rats
Source: PLoS One. 2013 Mar 27;8(3):e59712. doi: 10.1371/journal.pone.0059712 (PMC3609747; doi:10.1371/journal.pone.0059712)

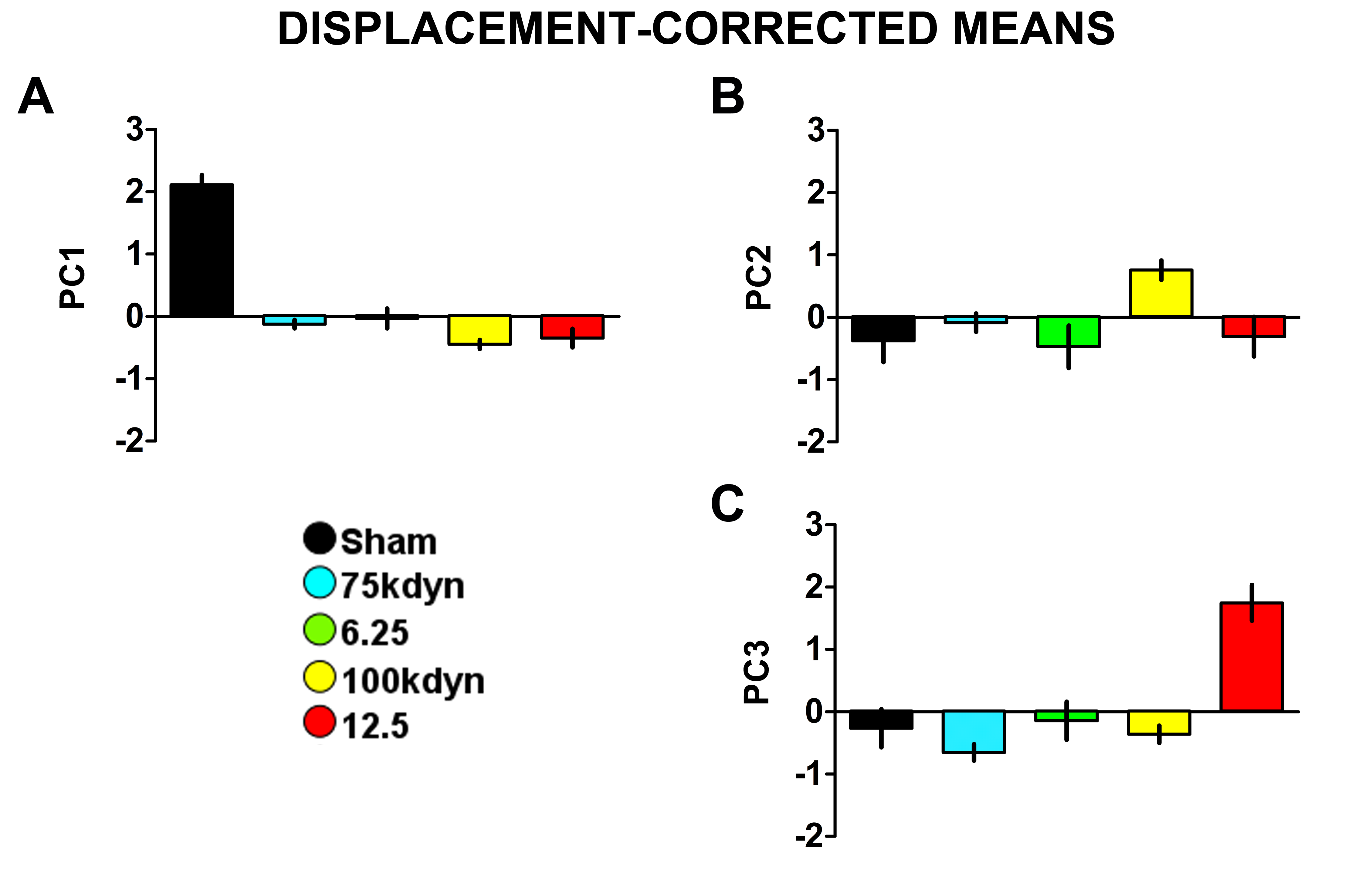

Supplement: Figure S2 — Effects of injury severity on PC1-3 after correcting for tissue displacement. Analysis of covariance (ANCOVA) indicated that tissue displacement was a significant covariate, for PC1 p<.05. However, correcting for tissue displacement did not alter the statistical significance of injury effects (compare to Fig. 4E–G). This suggests that differences across the injury devices were multivariate in nature and correcting for the biomechanical feature of displacement did not statistically account for the effects of injury. (TIF) [file pone.0059712.s002.tif]

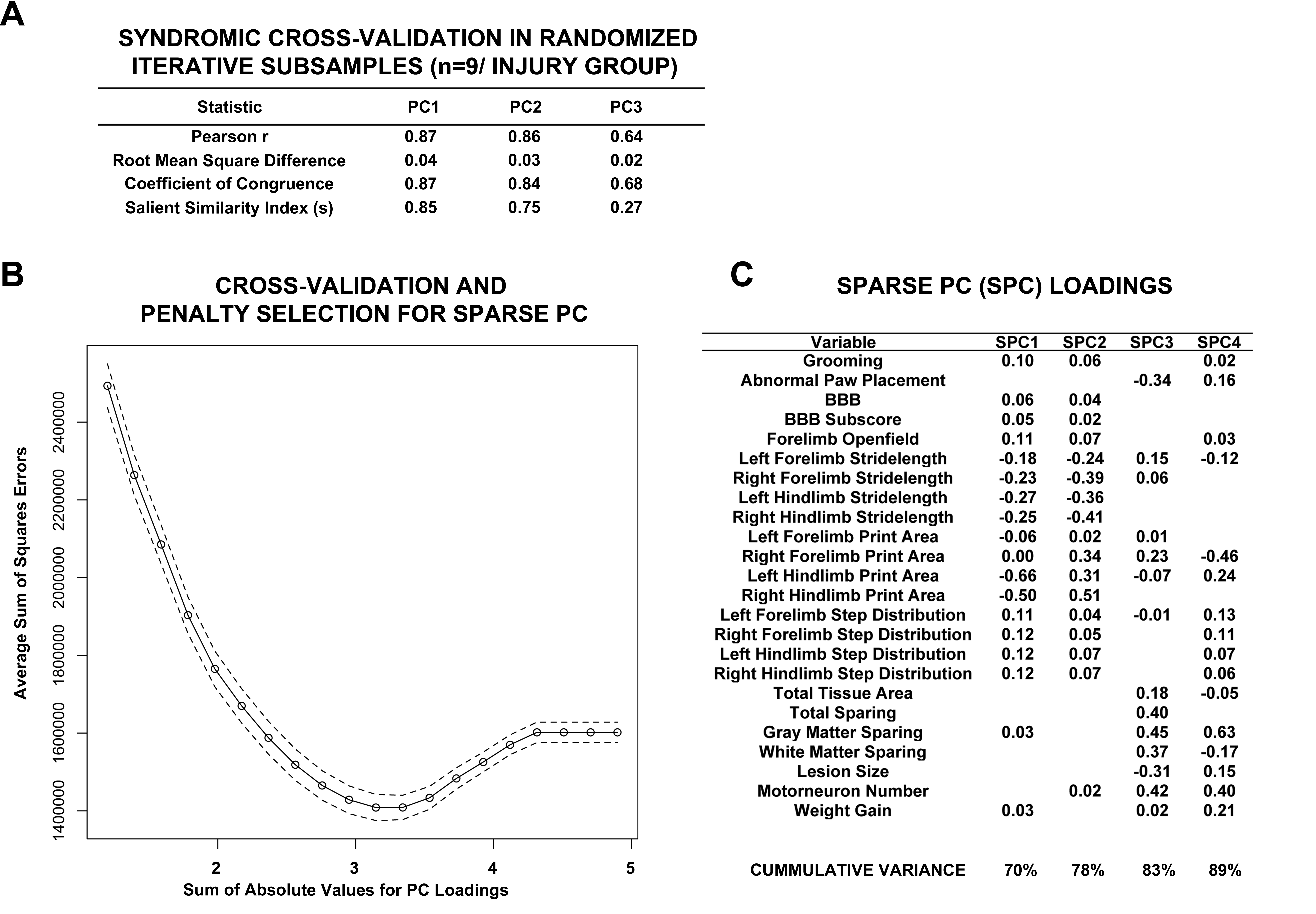

Supplement: Figure S5 — Cross-validation exercises using equalized n across groups and application of sparse PCA. A, Results from an iterative subsampling procedure used to homogenize group sizes (n = 9/injury condition) prior to PCA through 10 randomized subsampling iterations. PC pattern matching statistics comparing subsampled PC loading patterns to the loading pattern from the original dataset were averaged across iterations, revealing significant PC consensus in the subsampled populations. B–C, Application of a sparse PC algorithm with an L1 penalty to further evaluate PC consistency. B, Profile of cross-validated sums-of-squares errors as a function of extent of penalization suggested using a penalty value of 3. C, Sparse PC (SPC) loading matrix after penalty-induced shrinkage toward 0. Blanks indicate loadings of 0. Note that SPCA demoted PC1 moving it to PC3. Further examination of SPCA vs. other modern algorithms will be the subject of future in silico work using federated databases of SCI data that are currently under development. (TIF) [file pone.0059712.s005.tif]
